# Expression, purification and molecular analysis of the human ZNF706 protein

**DOI:** 10.1186/1480-9222-15-10

**Published:** 2013-09-23

**Authors:** Jucimara Colombo, Paola Jocelan Scarin Provazzi, Marilia Freitas Calmon, Lilian Campos Pires, Nathália Campos Rodrigues, Paulo Petl, Marcelo Andrés Fossey, Fátima Pereira de Souza, Fernanda Canduri, Paula Rahal

**Affiliations:** 1 Department of Biology, São Paulo State University - UNESP, CEP: 15054-000, São José do Rio Preto /SP, Brazil; 2 Institute of Chemistry of São Carlos, Department of Chemistry and Molecular Physics, University of São Paulo - USP, CEP: 13560-970, São Carlos /SP, Brazil; 3 Department of Physics, São Paulo State University - UNESP, CEP: 15054-000, São José do Rio Preto /SP, Brazil

**Keywords:** Circular dichroism, Cloning, HSPC038, Molecular modeling, Protein expression, ZNF706 protein

## Abstract

**Background:**

The *ZNF706* gene encodes a protein that belongs to the zinc finger family of proteins and was found to be highly expressed in laryngeal cancer, making the structure and function of ZNF706 worthy of investigation. In this study, we expressed and purified recombinant human ZNF706 that was suitable for structural analysis in *Escherichia coli* BL21(DH3).

**Findings:**

ZNF706 mRNA was extracted from a larynx tissue sample, and cDNA was ligated into a cloning vector using the TOPO method. ZNF706 protein was expressed according to the *E. coli* expression system procedures and was purified using a nickel-affinity column. The structural qualities of recombinant ZNF706 and quantification alpha, beta sheet, and other structures were obtained by spectroscopy of circular dichroism. ZNF706's structural modeling showed that it is composed of α-helices (28.3%), β-strands (19.4%), and turns (20.9%), in agreement with the spectral data from the dichroism analysis.

**Conclusions:**

We used circular dichroism and molecular modeling to examine the structure of ZNF706. The results suggest that human recombinant ZNF706 keeps its secondary structures and is appropriate for functional and structural studies. The method of expressing ZNF706 protein used in this study can be used to direct various functional and structural studies that will contribute to the understanding of its function as well as its relationship with other biological molecules and its putative role in carcinogenesis.

## Background

The ZNF706 gene encodes a protein that belongs to the zinc finger family of proteins. Zinc finger proteins are involving in endogenous gene expression control by binding to specific DNA sequences [1]. Zinc finger-containing transcriptional factors have previously been shown to participate in the MAPK signaling pathway regulation. These factors are among the principal methods of gene expression regulation on eukaryotic cells [2]. Moreover, the ZNF706 gene was associated with the Epidermal Growth Factor (EGF) to control the cell volume via the ICln gene [3]. ZNF proteins are now known to have additional activities such as the recognition of RNA and other proteins [4].

Despite the little information available about the biological functions of ZNF genes [5], some were indicated as participant in diseases or gene expression control. The transcriptional activity of activator protein 1 (AP1) was found to be enhanced by the ZNF445 gene [6]. The ZNF674, ZNF41 and its homologous ZNF81 are three X chromosome ZNF genes located in Xp11 and were associated with non-specific X-linked mental retardation [7,8]. The ZNF743 protein was found to be associated with methyl-CpG-binding protein 2 (MBD2) and was implicated in transcriptional and epigenetic controls [7].

The ZNF706 gene, which is located at 8q22.3, is a zinc finger gene family member. Currently, there are no studies that describe the more specific functions of this gene. However, it was observed that ZNF706 gene has a sex-specific gene expression pattern in human lymphoblastoid cell lines [8], and was found to be highly expressed in laryngeal cancer [9]. Likewise, copy number amplifications on chromosome 8 in cancer gastric samples were identified [10]. The gained regions detected in at least 25% of the samples were located at 8p11-q24 [10]. In addition, the ZNF706 gene was concordantly up-regulation in the analyzed gastric cancer samples [10]. High expression of the ZNF133 gene was also verified in head and neck cancer [11]. However, low expression of other zinc finger family members, such as ZNF185 [12,13], ZNF212 [14], ZNF262 [14] and ZNF273 [15] has already been observed in head and neck cancer. Thus, the zinc finger family genes can operate as enhancers or repressors of gene expression; depending on their action, they may have high or low expression levels in neoplastic tissues.

Structurally, the zinc finger proteins can be classified from the number and type of amino acids associated in zinc ion binding site, like the C_2_H_2_, C_2_HC, C_4_ ribbon, C_4_ GATA, C_6_, C_8_, C_3_HC_4_ ring finger and H_2_C_2_. The C_2_H_2_-type zinc finger proteins (of which ZNF706 is a member) have the most common DNA binding site and correspond to approximately 30% of all transcription factors in the human genome [16,17]. The C_2_H_2_-type zinc fingers fold into a ββα structure with the support of the zinc ion. Each finger typically recognizes 3–4 base pairs of DNA [17].

ZNF706 protein has 76 amino acids; however, no information on ZNF706 protein structure and function is available in the literature. In this work, we expressed, using a eukaryotic system, and purified the recombinant human ZNF706 human protein for structural analysis by molecular modeling and circular dichroism. Structural characterization may aid the study of the specific interactions of ZNF706 with other biological molecules such as inhibitors. This will contribute to the understanding of the specific function of ZNF706 and its putative role in carcinogenesis and to the design of new drugs.

## Results

**Figure 1:**
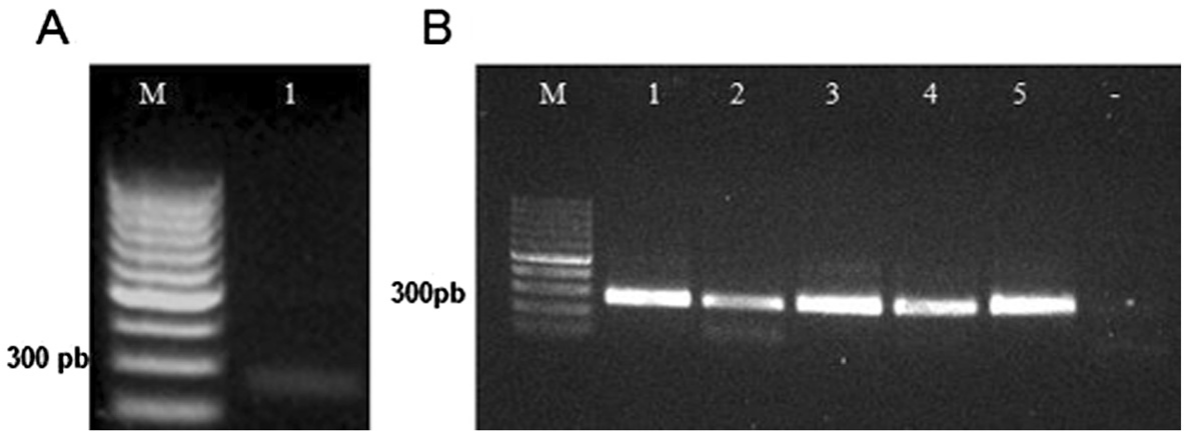
**Amplification of *ZNF706 *gene. A)** Agarose gel shows RT-PCR of the *ZNF706* gene*.* Lane M: molecular weight marker (Invitrogen, Grand Island, NY, USA); Lane 1: amplification of *ZNF706* gene (262 bp). **B)** Agarose gel shows amplification of the *ZNF706* gene cloned into pET101 expression vector in 5 plasmid preparations.

ZNF706 mRNA was extracted from a larynx tissue sample and cDNA was ligated into a cloning vector using the TOPO method (Invitrogen, Grand Island, NY, USA). Amplification of ZNF706 cDNA produced a fragment of approximately 262 bp (Figure 1A). After the cloning of this PCR product into the pET101 expression vector, five clones were verified using specific primers from the TOPO kit (Invitrogen, Grand Island, NY, USA). The PCR product was approximately 262 bp long (Figure 1B). Sequencing verified the identity of the sequence and the fidelity of cloning in clones 1 and 3 (data not shown).

Selected clones were transformed into *E. coli* BL21(DE3) competent cells and the protein expression was performed using the T7 system after induction with IPTG 1.5 mM IPTG (Sigma, St. Louis, MO, USA) for 16 hours.

**Figure 2:**
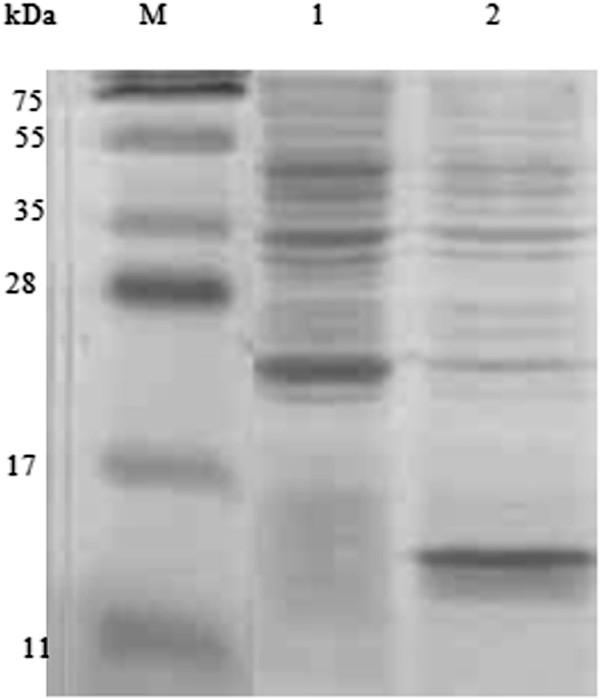
**Polyacrylamide gel SDS-PAGE 15% shows ZNF706 protein expression.** Lane M: molecular weight marker (PageRuler™ Prestained Protein Ladder - Fisher Scientific, Pittsburgh PA, USA); Lanes 1: no induction; Lane 2: 16 hours of induction with 1,5 mM IPTG.

**Figure 3:**
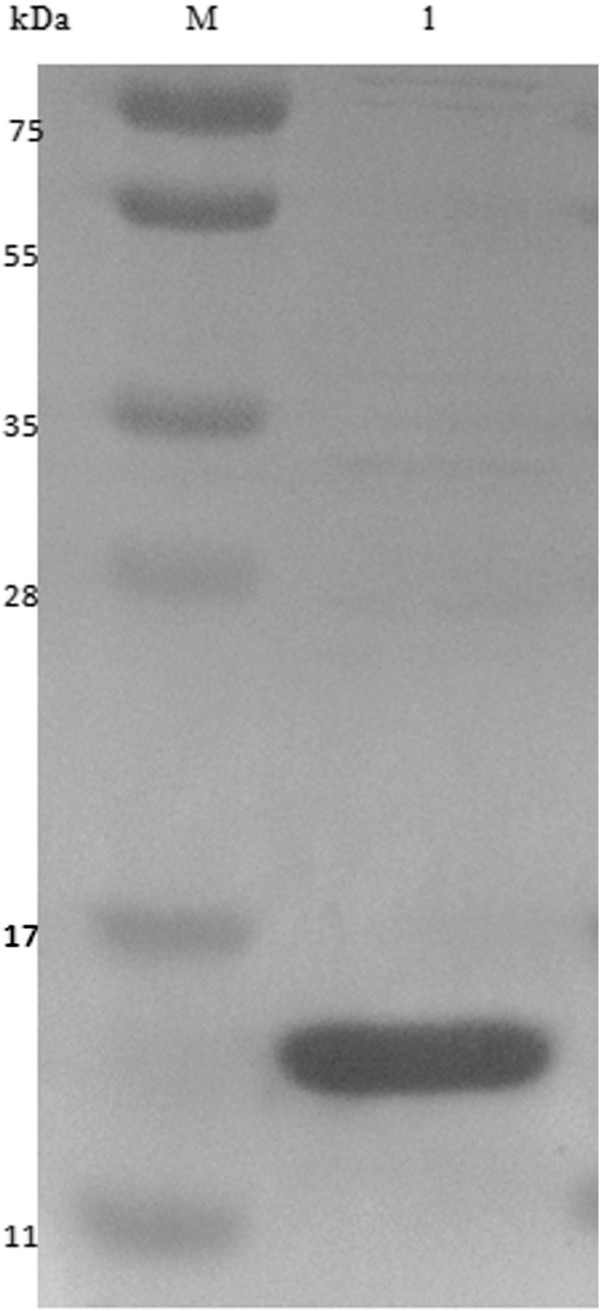
**Polyacrylamide gel SDS-PAGE 15% shows ZNF706 protein after purification and concentration procedures.** Line M: molecular weight marker (PageRuler™ Prestained Protein Ladder - Fisher Scientific, Pittsburgh PA, USA); Line 1: ZNF706 protein.

The SDS-PAGE electrophoresis showed the ZNF706 protein expression in a soluble fraction with a molecular weight of 12 kDa as determined by Coomassie Brilliant reagent (BIO-RAD, Hercules, CA, USA) (Figure 2). The protein was purified on a nickel-resin column (BIO-RAD, Hercules, CA, USA), and the fractions of ZNF706 protein could be visualized (Figure 3). ZNF706 protein has 76 amino acids and is 8.49 kDa in size. However, because the heterologous protein has a 6xHis tag and one start codon, the observed size is 12 kDa.

**Figure 4:**
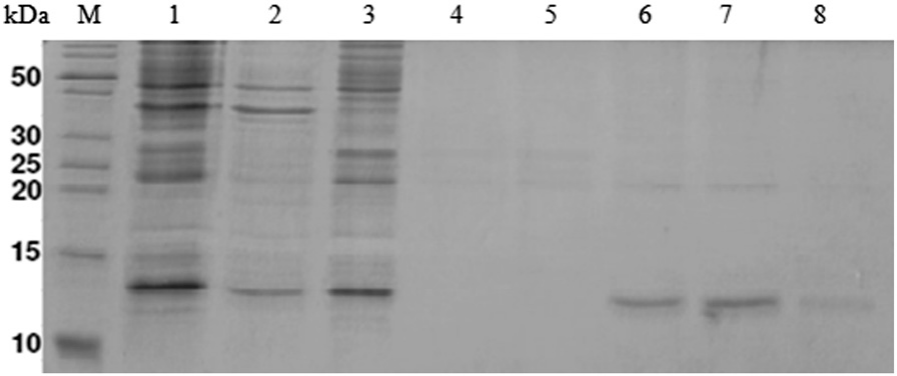
**Polyacrylamide gel SDS-PAGE 18% shows purification of the recombinant human ZNF706 protein by nickel-affinity chromatography.** Lane M: molecular weight marker (PageRuler™ Unstained Protein Ladder - Fisher Scientific, Pittsburgh PA, USA); Lane 1: crude extract. Lane 2: insoluble fraction. Lane 3: soluble fraction. Lane 4: Column washed with buffer plus imidazole 10 mM. Lane 5. Column washed with buffer plus imidazole 20 mM. Lanes 6-7. ZNF706 protein elution with buffer plus imidazole 250 mM. Lane 8: ZNF706 protein elution with buffer plus imidazole 500 mM.

After purification, the ZNF706 protein fractions were concentrated using an Amicon ultrafiltration cell system (MWC 3,000 Da) (Millipore, Billerica, MA, USA) (Figure 4). The ZNF706 concentration (3.0 mg) was obtained using the absorbance reading at 280 nm.

**Figure 5:**
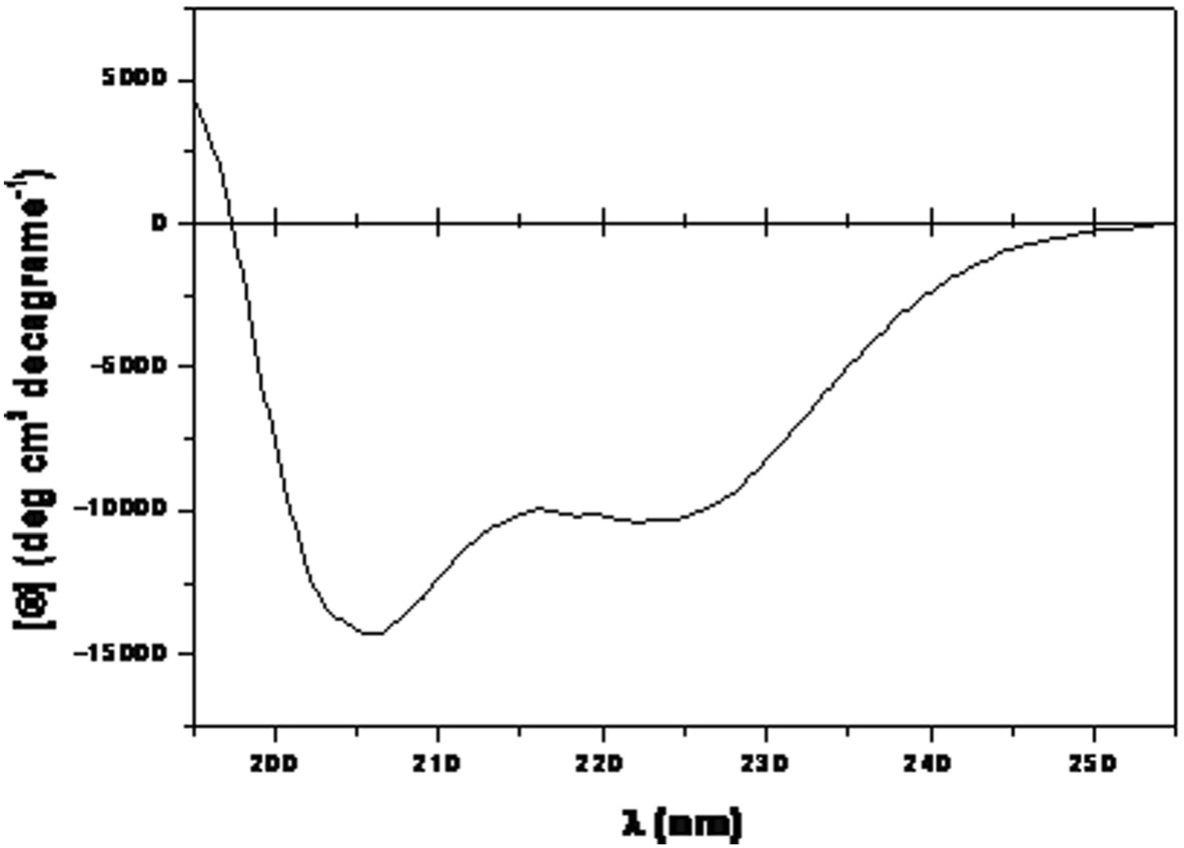
**Analysis of ZNF706 secondary structure by CD spectroscopy.** The CD spectrum of ZNF706 was acquired in 300 mM sodium phosphate, pH 8.0, at a concentration of 3,0 mg/ml.

The secondary structure content of ZNF706 was verified by CD spectroscopy (Figure 5). The CD spectrum showed that the protein contained a higher quantity of α-helical structures with a minimum ellipticity at 205 nm and at 222 nm. The secondary structure indicated by CDPro suite of the CD spectrum [18] shows that the protein is composed of α-helices (35%), β-strands (10%), turns (27%) and random coils (28%). The results obtained are in agreement with the expected structure for zinc finger proteins.

**Figure 6:**
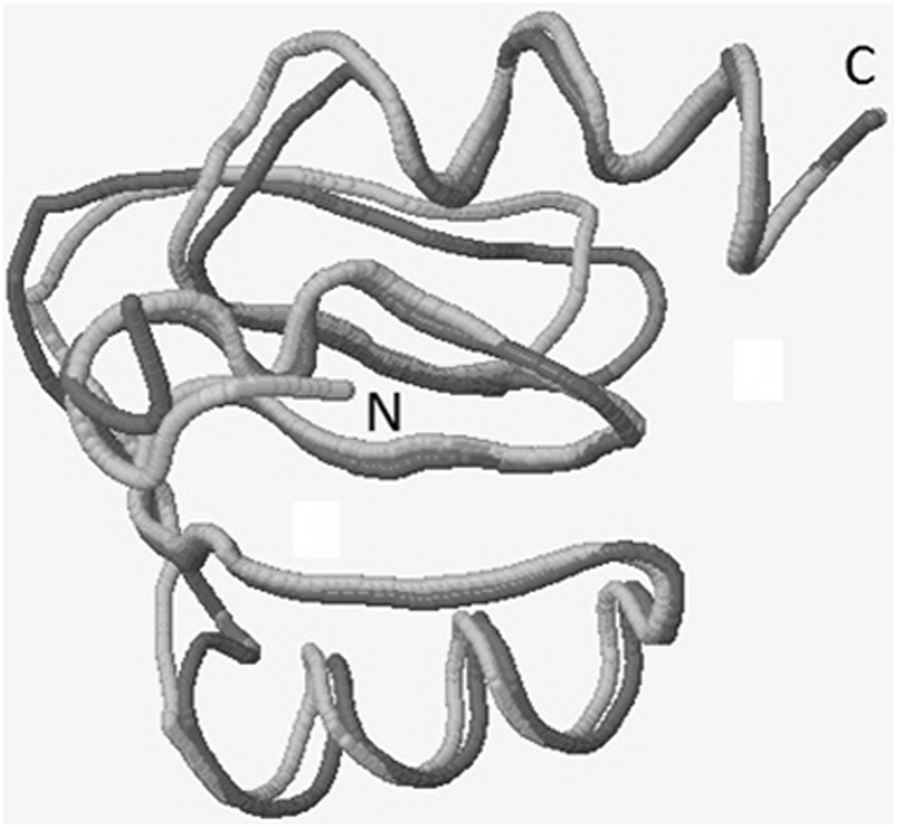
Superposition of main chain of HsZNF706 model (light gray) and 2GHF structure (dark gray).

The sequence alignment between HsZNF706 and the zinc-finger region of human Zinc- Fingers and Homeoboxes 1 (ZHX1) (PDB code 2GHF) showed 34.1% identity, with 41 amino acids aligned. HsZNF706 has 76 amino acid residues in the sequence, and the 2GHF template has 102 residues. Nine residues from the N-terminus of the target were removed. The structure generated by homology modeling [19,20] had an RMSD of 1.095 compared to the template; this estimate takes into account the Cα from the 65 superimposed residues (Figure 6). The global stereochemical quality [21] of the HsZNF706 protein model indicated that 98.3% of the amino acids were in the most allowed and additional allowed regions, with only 1.7% (Lys55) in the disallowed region. Structural analysis showed that the side chain of Lys55 is in contact with the solvent in a superficial conformation. The Procheck software [21] was used to calculate the G-factor average value and set to be -0.1. The Verify-3D [22] software analysis, revealed that 32.4% of the amino acids had an average value 3D- 1D score of >0.2, showing some compatibility with the HsZNF706 model. Fifty percent of the amino acids in the template had an average value 3D-1D score of >0.2.

**Figure 7:**
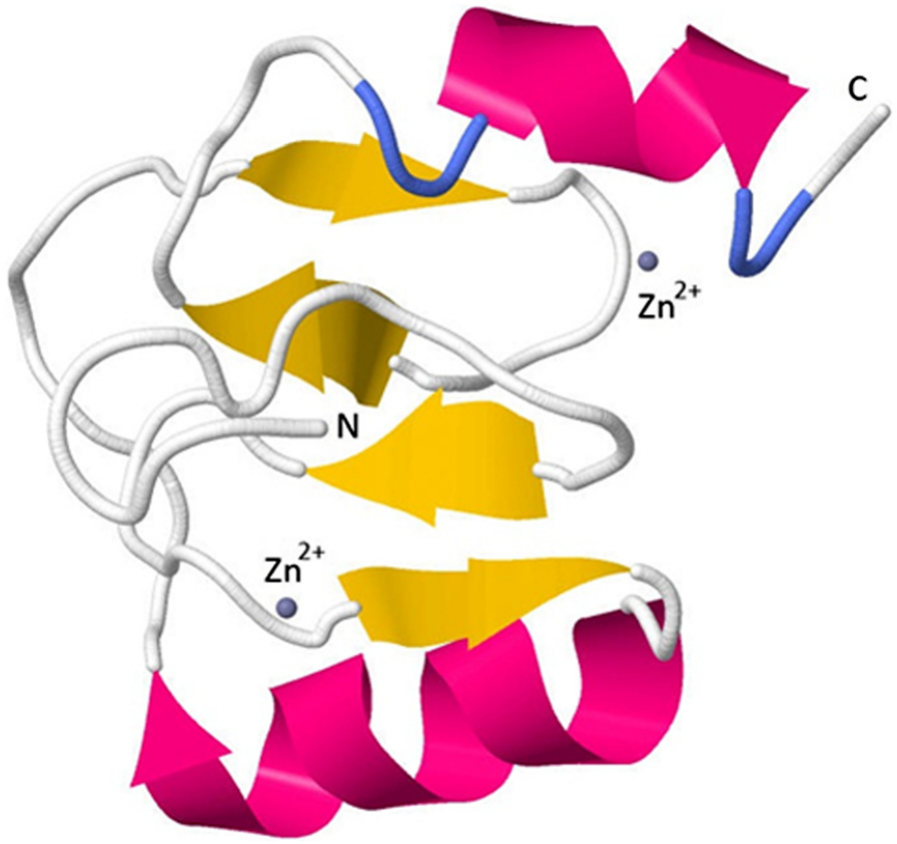
Secondary structure elements from HsZNF706 showing the zinc binding sites.

The HsZNF706 model consists of α-helices (28.3%), β-strands (19.4%) and turns (20.9%), compared to 25.8%, 16.1% and 20.4%, respectively, for the template. The structure is dominated by metal-ligand, heme and/or disulfide bridges, like other members of this small protein class, and is arranged in a β-β-α zinc finger fold. This model includes a simple fold consisting of the N-terminal β-hairpin and C-terminal α-helical region; each part provides two zinc-coordinating residues. Using the partial structural model of HsZNF706, the binding sites of the zinc ion were determined (Figure 7). The first ion is near the N-terminal β-hairpin in a site formed mainly by neutral and hydrophobic residues, and the second ion is near the second β-hairpin and C-terminal α-helix (Figure 7) at 2.9 Angstroms from the Q56 side chain and 2.7 Angstroms from the E70 side chain.

The generated model presents two cysteine residues next to each other in the tridimensional conformation (Cys41 and Cys44), but not involved in a disulfide bridge due to the distance between them (the distances of two bonded sulfur atoms is known to be distributed between 1.8 - 2.0 Å with a highest frequency at about 2.0 Å in proteins, and the distance between the sulfur atoms in the model is more than 3 Å). The model has a conserved folding module in ZNFs, which is the most probable folding obtained. The crystallographic structure will confirm these data. The N-terminal 9 residues of the target were not considered in this prediction because they are not reflected in the template and can be considered a characteristic region of HsZNF706.

## Discussion

Zinc finger (ZNF) proteins play critical roles in cell growth, proliferation, apoptosis, and intracellular signal transduction [23]. The *ZNF706* gene cDNA is 2755 bp in length and encodes a putative protein of 76 amino acid residues. However, the function and structure of the ZNF706 protein were uncharacterized. The results presented here show that recombinant *ZNF706* gene was cloned and the protein was expressed in *E. coli* bacteria cell line and purified from the soluble fraction in a single procedure step.

In this work, we used circular dichroism and molecular modeling to examining the structure of ZNF706. Circular dichroism (CD) spectroscopy is an important methodology used in structural biology for quantifying secondary structure content, conformational changes due to drug binding, folding, interactions involving protein molecules and protein quality [24]. Our results suggest that human recombinant ZNF706 keeps its secondary structures and is able to perform functional and structural studies. The agreement between the secondary structures predicted by molecular modeling and the secondary structure obtained by (CD) spectroscopy increases our confidence in the accuracy of the ZNF706 model. However, the N-terminal region of ZNF706 protein could not be resolved because there is no suitable template deposited in public databases. This region may be unique to the ZNF706 protein and therefore needs to be resolved. This problem reinforces the need for experimental structural studies involving techniques such as crystallography to solve the structure of the N-terminal region of this protein. Moreover, experimental determination of the three-dimensional structure will contribute to investigations of interactions of ZNF706 with ligands.

The knowledge of the Human ZNF706 protein structure is important for understanding the mechanisms of its interactions with other molecules and allowing structure-aided drug design [25,26]. The interaction between DNA target sequence and the zinc-finger proteins motivated the design of specific zinc finger peptides that can be used for medical and biochemical interaction studies [27,28].

An example of the application of these custom zinc finger proteins involves several kinds of methyltransferases, which have been linked to zinc-finger motifs and analyzed in vitro and in vivo conditions. This targeted DNA methylation allows artificial gene silencing and can be used to prevent an overexpressed gene in cancer by adding a methyl group to the target gene. Targeted DNA methylation was demonstrated to be effective at repressing Herpes Simples Virus type I (HSV-1) infection in cell culture [29]. Several groups have tested zinc fingers to inhibit the replication of integrated HIV [30,31] and high-risk HPV type 18, which is involved in cervical cancer [32]. Thus, custom zinc finger proteins can be engineered to control the expressions of target genes and have therapeutic potential for the study and treatment of diseases. Moreover, with further advances in zinc finger protein research, it may become possible to construct synthetic cell networks and control high-level vital functions [17].

The ZNF706 protein structural data obtained in this work by circular dichroism and molecular modeling can contribute to the understanding of the protein’s function, the relationships to other biological molecules and its possible role in carcinogenesis. Moreover, it represents the first step towards a future customized ZNF706 peptide that could act on cellular signaling networks and establishes the relationship between ZNF706 and diseases such as cancer.

## Conclusion

In this work, we used circular dichroism and molecular modeling to examine the structure of ZNF706. Our results suggest that recombinant human ZNF706 keeps its secondary structure and is appropriate for functional and structural studies. The agreement between the secondary structures predicted by molecular modeling and the secondary structure obtained by (CD) spectroscopy increases our confidence in the accuracy of the ZNF706 model. The data obtained in this work can contribute to the understanding of the protein’s function, relationships to other biological molecules and its possible role in carcinogenesis allowing structure-aided drug design. Moreover, it represents the first step towards a future customized ZNF706 peptide that could act on cellular signaling networks and establishing the relationship between ZNF706 and diseases such as cancer.

## Methods

### Reverse transcription reaction and ZNF706 cDNA cloning

For the Reverse Transcription (RT-PCR) procedure, total RNA was extracted from larynx tumor tissue samples using Trizol reagent (Invitrogen). The full-length ZNF706 cDNA was performed using the following oligonucleotide primers: Forward, **CATCACCAT***CAG*ATGGCTCGTGGACAG; Adapt F, CACC**ATG***AAA***CATCATCACCATCACCAT***CAG* and Reverse, **TTA**TGCCTGAACATCAGCTA, which were constructed using the GenBank ZNF706 cDNA sequence No. NM_016096.3 as the reference. The N-terminal polyhistidine tag used for recombinant protein purification is shown in bold. The protease recognition site for removal of the N-terminal polyhistidine tag is shown in italics. The nucleotides CACC are the overhang sequence for binding to pET101 (Invitrogen).

The ZNF706 cDNA (231 bp) was amplified using the constructed set of primers and standard PCR conditions with Elongase (Invitrogen). After checking the amplification on an agarose gel, nested PCR with the product from the first PCR and Adapt F and Reverse primers was performed using the same amplification conditions. The amplification products were checked on an agarose gel and consisted of a specific band of approximately 262 bp, corresponding to the expected size of the ZNF706 gene (231 bp) plus the 31 bp of the Adapt F primer.

### Subcloning in *E. coli* TOP10

After purification with the Qiagen Purification Kit, the amplified fragment was inserted into the pET101 expression vector following the Championg pET Directional TOPO Expression Kit manufacturer’s instructions (Invitrogen). pET101::ZNF706 was transformed into E.coli DH5alpha competent cells. Several colonies were collected from Luria-Bertani (LB) plates supplemented with ampicillin (50 μg/ml) and then inoculated into 5 ml of LB medium containing ampicillin (50 μg/ml). The culture was incubated for 16 hours at 37°C. Recombinant plasmid was extracted with a PureLink plasmid mini prep kit (Invitrogen). The fidelity of the cloned product was verified by sequencing using the specific primers provided in the TOPO kit.

### ZNF706 protein expression

The expression plasmids pET101::znf706 were transformed into *E. coli* BL21(DE3) competent cells. After selection on LB agar plates (ampicillin - 50 μg/mL), recombinant colonies were inoculated into 5 mL of LB medium supplemented with ampicillin (50 μg/mL). To test the optimal condition for ZNF706 protein expression, *E. coli* BL21 (DE3) cells carrying pET101::znf706 were grown in the presence of IPTG (isopropyl β-D-thiogalactoside) at different concentrations: 1.5 mM, 1.0 mM, 0.75 mM and 0.5 mM. After centrifugation, the cells were re-suspended in lysis buffer containing 100 mM NaCl, 10 mM Tris and 50 mM NaH_2_PO_4_, at pH 8.0) and disrupted by sonication (eight short bursts of 15 s). The proteins were separated by SDS-PAGE electrophoresis and the bands were visualized using Coomassie Blue reagent.

### Large scale expression and purification

A total of 20 mL of LB cell culture containing the recombinant plasmid was diluted in 2 L of LB supplemented with ampicillin (50 μg/mL). The culture was incubated at 37°C and 240 rpm until the cells achieve OD_600 _= 0.6. At this point, the ZNF706 expression was induced with 1.5 mM of IPTG following incubation at 37°C for 16 hours. The culture cell was submitted to centrifugation and the harvested cells were re-suspended in 20 mL of lysis buffer (100 mM NaCl, 10 mM Tris and 50 mM NaH_2_PO_4_, pH 8.0). To lyse the cells, the samples were incubated on ice with lysozyme (0.5 mg/mL) for 30 min. After sonication (eight short bursts, with 15 s) the lysate was submitted to centrifugation for 30 min at 14,000 rpm and 4°C. The supernatant was loaded onto a nickel-affinity column (BIO-RAD; Profinity™ IMAC Ni-Charged Resin; 4 mL) pre-equilibrated with purification buffer containing 50 mM NaH_2_PO_4_ and 300 mM NaCl, at pH 8.0. After washing, bound proteins were eluted with an imidazol gradient (20 mM, 50 mM, 250 mM and 500 mM imidazol in purification buffer). Fractions containing ZNF706 were pooled, concentrated and dialyzed using an Amicon ultrafiltration cell (MWC 3,000 Da). The protein was analyzed by SDS-PAGE electrophoresis and visualized using Coomassie Brilliant Blue reagent.

The recombinant ZNF706 quantification was obtained by direct absorbance readings at 280 nm.

### Circular dichroism

Circular dichroism (CD) spectroscopy was realized in a Jasco 710 spectropolarimeter at 25°C. The CD spectra was measured with a protein concentration of 3.0 mg/mL in phosphate buffer pH 8.0, (300 mM), using a 0.05 mm optical length. The spectra were collected from 195 to 255 nm. The results were converted to mg/ml ellipticity units, [Φ] (deg cm2 decagram-1), and the alpha, beta sheet, and other structures were verified using CDPro software package [18].

### Molecular modeling

The program MODELLER 9v8 was used to model the HsZNF706 structure. This program is an automated approach for homology or comparative modeling by satisfaction of spatial restraints [19,20]. The molecular modeling methodology began with the alignment of the target protein to be determined (HsZNF706) with the templates (related known three-dimensional protein structures). The template was a human Zinc fingers and homeoboxes protein 1 (ZHX1) (PDB code 2GHF) solved by Nuclear Magnetic Resonance (NMR), which has 34.1% identity and an overlap of 41 amino acids with the HsZNF706 sequence. The alignment among the amino acid sequence was the input of the software and a three-dimensional model of the target protein sequence was the output. The energy terms and spatial restraints enforcing proper stereochemistry [33] were combined into an objective function. The resulting ZNF protein models were generated by optimization of the objective function in a Cartesian coordinate system. The optimization was performed with the variable target function method [34] by employing method of conjugate gradients and simulated annealing. A total of 300 models were obtained, and the final model was selected based on the stereochemical quality.

The overall stereochemical quality of the HsZNF706 model was based on the following analyses: 1) a Ramachadram plot obtained using the program PROCHECK [21], 2) the rmsd from the distance of secondary structure alignment calculated using PDBeFold [http://www.ebi.ac.uk/msd-srv/ssm/], 3) the G-factor value calculated for the torsion angles (phi-psi, chi1-chi2, chi-1, chi-3, chi-4 and omega values for each amino acid residue) and covalent geometry (for the main-chain bond lengths and bond angles; calculated using PROCHECK) [21] and 4) the compatibility of the protein model with its sequence, calculated using Verify -3D software and a three-dimensional profile [22].

## Abbreviations

ZNF706: Zinc finger 706; LB: Luria-bertani medium; CD: Circular dichroism; NMR: Nuclear magnetic resonance; RMSD: Root mean square deviation; PDB: Protein data bank; RT-PCR: Reverse transcriptase-polymerase chain reaction.

## Competing interests

The authors have no conflict of interest.

## Authors’ contributions

Experiments were performed by JC, PJSP, LCP, NCR, PP, MF and FPS. JC, PJSP, MFC, FC, PFS and PR wrote and edited the text and all approved the final manuscript.
